# Antidiabetic and Cardioprotective Effects of Pharmacological Inhibition of GRK2 in db/db Mice

**DOI:** 10.3390/ijms20061492

**Published:** 2019-03-25

**Authors:** Ersilia Cipolletta, Jessica Gambardella, Antonella Fiordelisi, Carmine Del Giudice, Eugenio Di Vaia, Michele Ciccarelli, Marina Sala, Pietro Campiglia, Enrico Coscioni, Bruno Trimarco, Daniela Sorriento, Guido Iaccarino

**Affiliations:** 1 Department of Advanced Biomedical Sciences, “Federico II” University of Naples, 80131 Napoli, Italy; ecipolletta@tiscali.it (E.C.); gambardellajessica@gmail.com (J.G.); antonellafiordelisi@gmail.com (A.F.); degiudicec@gmail.com (C.D.G.); eugenio.divaia@unina.it (E.D.V.); trimarco@unina.it (B.T.); daniela.sorriento@unina.it (D.S.); 2 Department of Medicine, Surgery and Dentistry, University of Salerno, 84081 Baronissi, Italy; mciccarelli@unisa.it; 3 Department of Pharmacy, University of Salerno, 84084 Fisciano, Italy; marina.sala@unisa.it (M.S.); pcampiglia@unisa.it (P.C.); 4 AOU San Giovanni di Dio e Ruggi d’Aragona, 84131 Salerno, Italy; coscionienrico@gmail.com

**Keywords:** kinase, signaling, insulin sensitivity, heart failure, kinase inhibitor

## Abstract

Despite the availability of several therapies for the management of blood glucose in diabetic patients, most of the treatments do not show benefits on diabetic cardiomyopathy, while others even favor the progression of the disease. New pharmacological targets are needed that might help the management of diabetes and its cardiovascular complications at the same time. GRK2 appears a promising target, given its established role in insulin resistance and in systolic heart failure. Using a custom peptide inhibitor of GRK2, we assessed in vitro in L6 myoblasts the effects of GRK2 inhibition on glucose extraction and insulin signaling. Afterwards, we treated diabetic male mice (db/db) for 2 weeks. Glucose tolerance (IGTT) and insulin sensitivity (ITT) were ameliorated, as was skeletal muscle glucose uptake and insulin signaling. In the heart, at the same time, the GRK2 inhibitor ameliorated inflammatory and cytokine responses, reduced oxidative stress, and corrected patterns of fetal gene expression, typical of diabetic cardiomyopathy. GRK2 inhibition represents a promising therapeutic target for diabetes and its cardiovascular complications.

## 1. Introduction

Metabolic diseases represent the fastest growing epidemic worldwide, associated with a significant increase in comorbidities and healthcare costs [1]. Type 2 diabetes mellitus (T2DM) shares an intimate relationship with altered metabolism through the development [2] of insulin resistance (IRES). The investigation of the mechanisms of T2DM is crucial to refining therapeutic strategies to reduce morbidity and mortality. The latter is due in the largest proportion of T2DM to cardiovascular disease and heart failure. In particular, diabetic patients present with structural and functional abnormalities in the heart referred to as diabetic cardiomyopathy, a condition that progresses from heart failure with preserved ejection fraction (HFpEF) towards heart failure with reduced ejection fraction (HFrEF) [3,4]. Interestingly, traditional therapies of diabetes are not able to interfere with this feature, and in some cases, they even promote the progression of heart failure and are associated with increased morbidity and mortality [4]. The introduction of antidiabetic drugs targeting a novel mechanism of blood glucose regulation has shown that this fatal union is not obligatory. Therefore, the quest is open for antidiabetic drugs that, at the same time, can ameliorate structure and function in the diabetic heart.

The G protein-coupled receptor kinase type 2 (GRK2) is involved in the regulation of many pivotal cell functions and is a key player in human health and diseases [5]. GRK2 is the long-recognized mechanism for agonist-activated G protein-coupled receptor (GPCR) desensitization and internalization [5,6]; increased GRK2 levels and/or activity have important implications in many cardiovascular conditions, such as myocardial ischemia, hypertrophy, and hypertension [7,8]. In recent years, the identification of a large “interactome” extended the role of this kinase to the regulator of cell metabolism [9,10,11,12]. In fact, GRK2 regulates insulin signaling [13,14], through serine phosphorylative events [14,15]. Excessive GRK2 up-regulation inhibits insulin signaling and glucose extraction [14] due to a time-dependent insulin-stimulated association of GRK2 with Insulin Receptor Substrate 1 (IRS1), leading to IRS1 serine phosphorylation and inactivation [14]. In this sense, GRK2 activity inhibition improves insulin sensitivity, providing a new therapeutic target for the treatment of IRES and T2DM [14,15].

Interestingly, GRK2 inhibition can correct left ventricle (LV) dysfunction in several models of heart failure, due to multiple accountable mechanisms, from the relief of beta-adrenergic receptor desensitization to improved glucose metabolism [16]. We have recently demonstrated in a model of HFpEF, the spontaneously hypertensive rat, that GRK2 causes left ventricle hypertrophy through the activation of the NF-κB dependent transcription pathway [17]. 

A series of small molecules, putative inhibitors to GRK2, are already available with ranging selectivity [18,19,20,21,22]. On the other hand, we have developed a novel strategy of inhibiting GRK2, based on peptides targeting the aminoacidic sequence of the kinase named HJ loop [23,24], which, by preventing the conformational shifts needed for kinase substrate interaction, inhibits the kinase in a selective fashion. In particular, we have identified KRX-C7, obtained through side chain cyclization of the linear peptide derived from fragment 383–390 of the HJ loop of GRK2, which showed more stability, selectivity, and inhibitory activity compared to parent linear molecules in cellular models [23]. 

The aim of this study is to produce the proof of concept in vitro and in vivo that GRK2 inhibition through KRX-C7 represents an effective therapeutic approach for the treatment of T2DM with positive effects on diabetic cardiomyopathy.

## 2. Results

### 2.1. KRX-C7 Inhibits GRK2 Kinase Activity in L6 Myoblast

**Figure 1:**
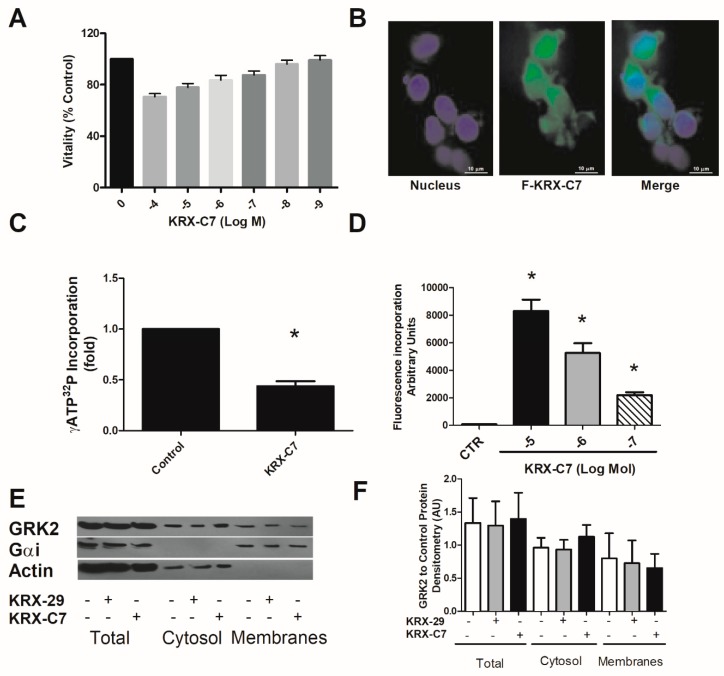
Biological properties of KRX-C7. (**A**) Effects of KRX-C7 on cell viability are expressed as the percentage of viable cells relative to the control (untreated cells). (**B**) The ability of KRX-C7 to penetrate cell membranes of serum starved L6 myoblasts was assessed through the fluorescent peptide (F-KRX-C7, 10^−6^ mol/L). Images were obtained by fluorescent microscopy. (**C**) Quantification of three experiments measuring fluorescence incorporation of the cells incubated with either 10 µM or 1 µM. (**D**) KRX-C7 inhibition of GRK2 activity in L6 myoblast cytosolic fractions (100–150 µg of protein) tested by ^y32^P-ATP incorporation on rod outer substrates (ROS). (**E**) Effects of GRK2 inhibitors on kinase subcellular localization in L6 myoblasts; KRX-29 (linear peptide, 10^−6^ mol/L); KRX-C7 (10^−6^ mol/L) (**F**). Dendsitometric analysis of the effects of GRK2 inhibitors on kinase subcellular localization in L6 myoblasts; KRX-29 (linear peptide, 10^−6^ mol/L); KRX-C7 (10^−6^ mol/L). Each data point in all graphs represents the mean ± SEM of three independent experiments. * *p* < 0.05 vs. control.

To investigate the possible cytotoxicity of KRX-C7 in L6 myoblasts, we measured cell viability. As shown in Figure 1A, KRX-C7 up to 1 × 10^−6^ M did not significantly modify cell viability. Then, we confirmed the ability of KRX-C7 to penetrate the cell membrane by fluorescence (Figure 1B,C). Using cytosol extracts to test GRK2 activity, we demonstrated the ability of KRX-C7 to inhibit the GRK2 activity in L6 myoblasts (Figure 1D). Finally, we reported that KRX-C7, or a parental linear compound (KRX-29), does not affect GRK2 membrane localization (Figure 1E).

### 2.2. KRX-C7 Improves Insulin Sensitivity

**Figure 2:**
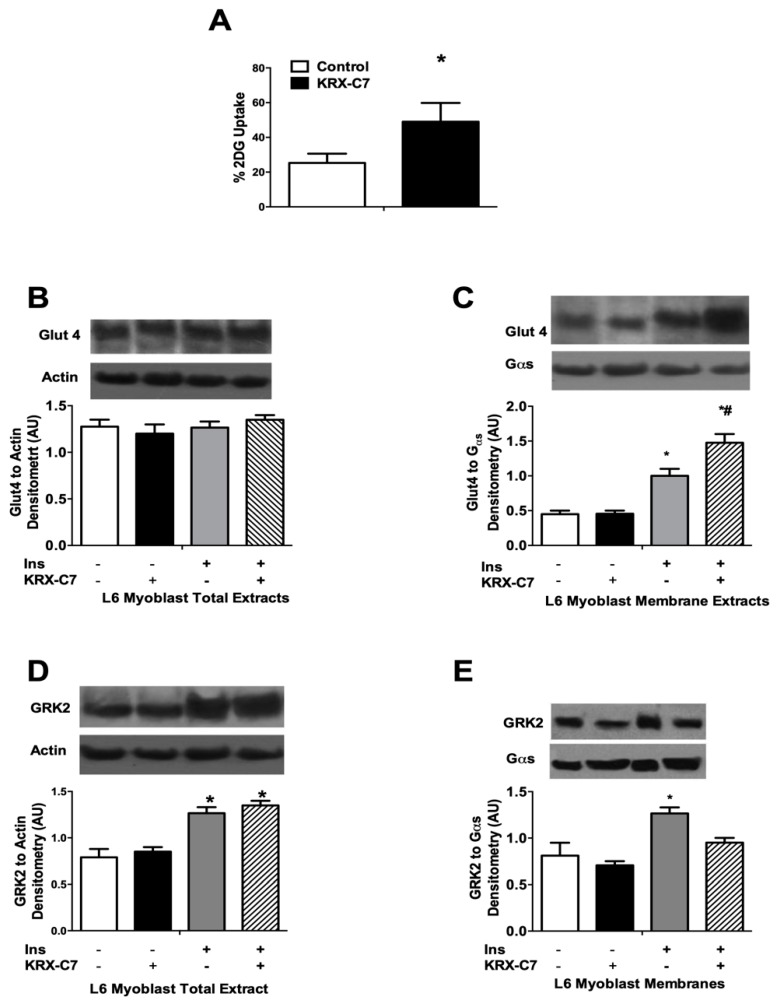
Effects of KRX-C7 on glucose extraction and GRK2 expression in L6 myoblasts. (**A**) Effects of KRX-C7 on basal and insulin (Ins) -induced glucose uptake determined by ^3^[H]-2-Deoxyglucose (2-DOG). Results are expressed as mean ± SEM of three independent experiments. * *p* < 0.05 vs. Ctr. (**B**) Effects of KRX-C7 on glucose transporter 4 (Glut4) expression. Densitometry of GLUT4 was corrected for actin. (**C**) Effects of KRX-C7 on Glut4 membrane translocation. Densitometry of GLUT4 was corrected for Gαs. (**D**) Effects of KRX-C7 on insulin induced on GRK2 expression in L6 myoblasts. (**E**) Effects of KRX-C7 on insulin induced on GRK2 translocation to cell membranes in L6 myoblasts. For all graphs, densitometric analysis is expressed after normalization for appropriate control protein. Data points represent the mean ± SEM of three independent experiments. * *p* < 0.05 vs. Ins−; # *p* < 0.05 vs. Ins+.

To verify if KRX-C7 sensitizes the response to insulin, we evaluated insulin-induced glucose extraction in L6 cells. It was shown that KRX-C7 enhances insulin-mediated glucose uptake (Figure 2A). Mechanistically, KRX-C7 does not affect Glut 4 expression (Figure 2B), but, rather, it enhances its translocation to the plasma membrane after insulin (Figure 2C). As expected, insulin induces the increase of GRK2 total expression, and KRX-C7 does not modify total levels (Figure 2D), while attenuates insulin-induced membrane localization (Figure 2E) of the kinase.

**Figure 3:**
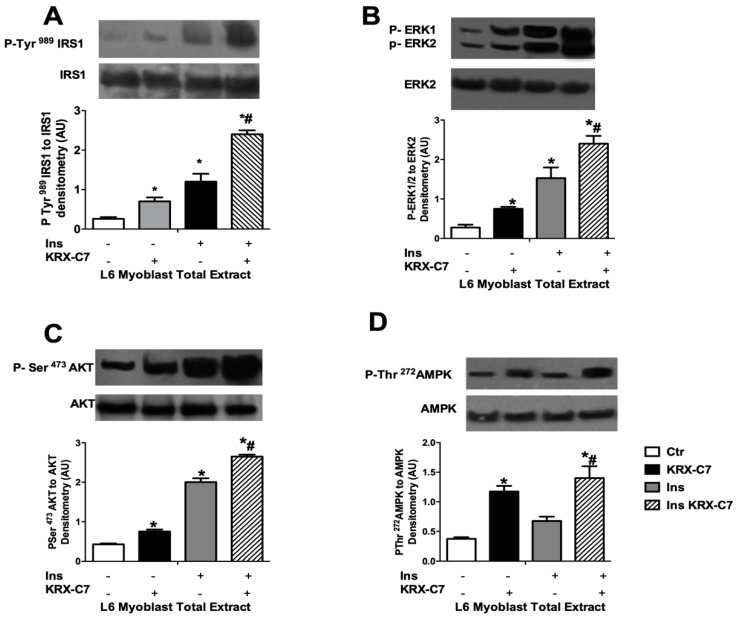
KRX-C7 ameliorates insulin signaling in L6 myoblasts. (**A**) Effects of KRX-C7 on insulin mediated IRS1 phosphorylation in L6 myoblasts. IRS1 phosphorylation densitometry was corrected by total IRS1 densitometry. (**B**–**D**) Total cellular samples were analyzed by WB for Phospho-Ser473AKT, Phospho-tyrosine 42/44 ERK, and Phospho-Ser272-AMPK with specific antibodies. Intensity on WB was quantified by densitometric analysis. Phosphorylation densitometry was corrected by the corresponding total protein densitometry. Data points in all graphs represent the mean ± SEM of three independent experiments. * *p* < 0.05 vs. Ins−; # *p* < 0.05 vs. Ins+.

Next, we examined the effects of KRX-C7 on insulin signaling. In particular, we observed that exposure to KRX-C7 increased insulin-induced IRS1 tyrosine phosphorylation (Figure 3A) as well as AKT (Figure 3B), and ERK (Figure 3C) phosphorylation. On the other hand, we observed that GRK2 inhibition potentiated insulin-mediated phosphorylation of AMP-activated protein kinase (AMPK) (Figure 3D), which is widely regarded as a key regulator of mitochondrial biogenesis. These data suggest that GRK2 is also involved in cellular energy management [10,25,26]. 

### 2.3. KRX-C7 Improves Insulin Sensitivity in db/db Mice

**Figure 4:**
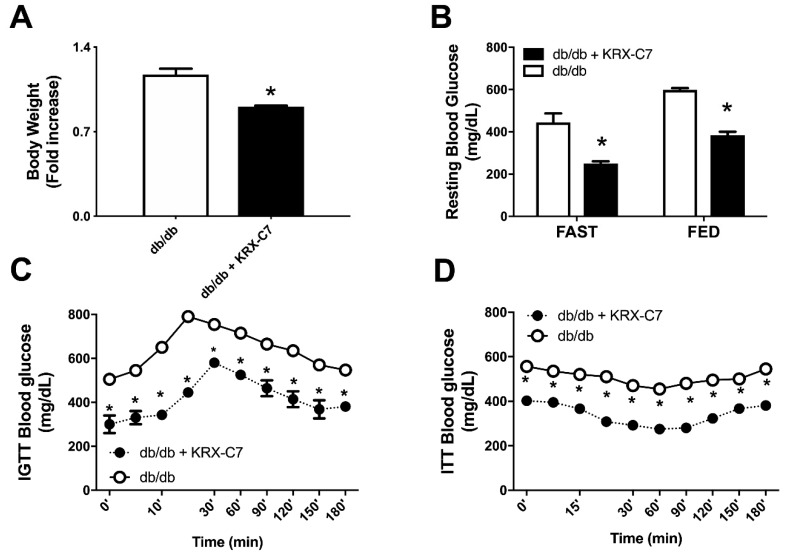
KRX-C7 improves insulin sensitivity in db/db mice. (**A**) Effects on body weight of chronic infusion of KRX-C7 (db/db KRX-C7). (**B**) Effects of KRX-C7 treatment on blood glucose levels both in fasted and fed conditions in KRX-C7 treated or control db/db mice. (**C**) Effects of KRX-C7 in db/db on blood glucose levels during an intraperitoneal glucose tolerance test. (**D**) Effects of KRX-C7 in db/db on blood glucose levels during an intraperitoneal insulin tolerance test. Values in each graph are the means ± SEM, *n* = 6 animals per group. ○: db/db; ●: db/db + KRX-C7; * *p* < 0.05 vs. db/db.

KRX-C7 treatment reduced body weight (Figure 4A) and systolic blood pressure (118 ± 4 vs. 137 ± 3 mmHg, *p* < 0,05) compared to controls. Furthermore, KRX-C7 treatment ameliorated blood glucose levels both in fasted and fed conditions (Figure 4B). The improvement of insulin sensitivity induced by the administration of the GRK2 selective inhibitor was confirmed by amelioration of glucose tolerance in IGTT (Figure 4C) and ITT, which showed a greater decrease of blood glucose levels in comparison with age-matched controls (Figure 4D).

### 2.4. Effects of KRX-C7 Treatment on Insulin Mediated Glucose Transport Ex Vivo

**Figure 5:**
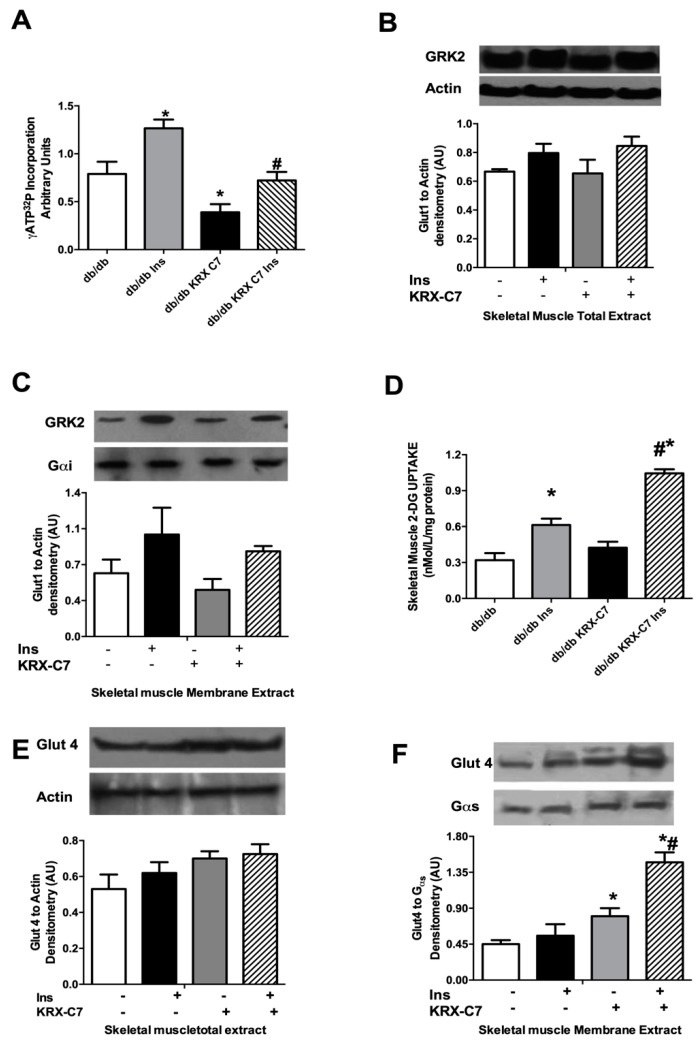
Effects of KRX-C7 treatment on insulin-mediated glucose transport ex vivo. (**A**) GRK2 activity in cytosols extracted from skeletal muscle was assessed by [^γ32^P]-ATP incorporation into ROS. (**B**) Effects of KRX-C7 on GRK2 levels in the total extract from femoral quadriceps muscles analyzed by WB for GRK2. GRK2 levels were averaged and normalized to actin. (**C**) Effects of KRX-C7 on GRK2 levels in membrane extracts from femoral quadriceps muscles, analyzed by WB and corrected by Gαs densitometry. (**D**) Isolated quadriceps femoral muscles from db/db and db/db+KRX-C7 mice were used to perform basal and insulin-stimulated [3H]-2-Deoxyglucose (2-DOG) transport. (**E**) Glut4 expression on skeletal muscles membrane extracts from db/db and db/db+KRX-C7 were analyzed by Western blot (WB) and normalized to Gαs densitometry. (**F**) Glut4 expression in whole extracts of quadriceps femoral muscles were analyzed by WB and normalized for actin. Data points in all graphs represent the mean ± SEM of three independent experiments. * *p* < 0.05 vs. db/db. # *p* < 0.05 vs. db/db+KRX-C7.

To further clarify the effects of the GRK2 inhibitor on insulin-mediated glucose disposal, we investigated glucose transport in isolated quadriceps femoral muscles from db/db mice. KRX-C7 reduced GRK2 activity in this tissue (Figure 5A) without any change in the total kinase expression (Figure 5B). KRX-C7 decreased insulin-mediated GRK2 membrane localization (Figure 5C).

Next, we evaluated the effects of KRX-C7 on glucose transport in skeletal muscle, showing improved [³H]-2-deoxyglucose uptake in femoral quadriceps (Figure 5D). The demonstration that the GRK2 inhibitor increases insulin sensitivity in db/db skeletal muscle was confirmed by the effects on insulin regulation of Glut4. We found that chronic treatment with GRK2 peptide inhibitor induced a significant increase of insulin-induced Glut4 membrane translocation (Figure 5E) without changes in Glut4 total protein levels (Figure 5F). These data suggest that treatment with GRK2 inhibitor increases insulin signaling in the skeletal muscle of db/db mice.

### 2.5. KRX-C7 Improves Insulin Signaling in db/db Mice

**Figure 6:**
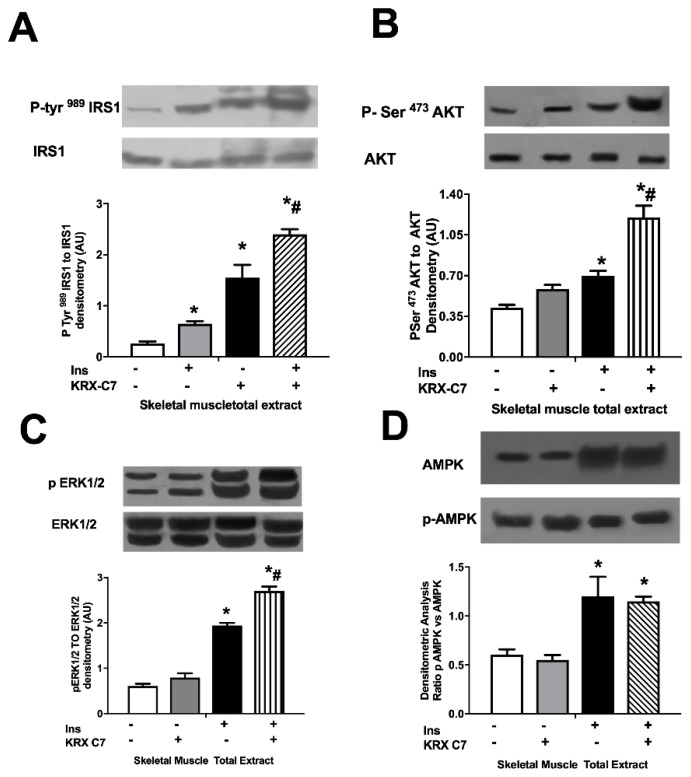
KRX-C7 improve insulin signaling in db/db mice. (**A**) Effects of KRX-C7 chronic infusion in the femoral quadriceps of db/db mice on insulin induced phospho-IRS1. (**B**) Effects of KRX-C7 chronic infusion in the femoral quadriceps of db/db mice on insulin induced Phospho-Ser473AKT. (**C**) Effects of KRX-C7 chronic infusion in the femoral quadriceps of db/db mice on insulin induced Phosphotyrosine 42/44 ERK. (**D**) Effects of KRX-C7 chronic infusion in the femoral quadriceps of db/db mice on insulin induced Phospho-Ser272-AMPK. For all graphs, densitometric analysis is expressed after normalization for appropriate control protein. Data points represent the mean ± SEM of three independent experiments. * *p* < 0.05 vs. Ins−; # *p* < 0.05 vs. Ins+.

We therefore examined the effects of KRXc7 administration on insulin signaling in skeletal muscle. Immunoblot analyses showed that the expression of insulin-signaling relevant proteins, such as IRS1 (Figure 6A), AKT (Figure 6B), ERK1/2 (Figure 6C), and AMPK (Figure 6D), was not modified by KRX-C7; the inhibitor, on the contrary, enhanced insulin-mediated tyrosine phosphorylation of IRS1 (Figure 6A), Serine 473 phosphorylation of AKT (Figure 6B), phosphorylation of ERK (Figure 6C), and AMPK phosphorylation (Figure 6D). These data demonstrate that GRK2 plays an essential role in diabetic muscles to regulate insulin signaling and insulin-mediated glucose homeostasis and its inhibition can restore insulin sensitivity in metabolically active tissues. 

### 2.6. KRX-C7 Reduces Cardiac Inflammation and Oxidative Stress

**Figure 7:**
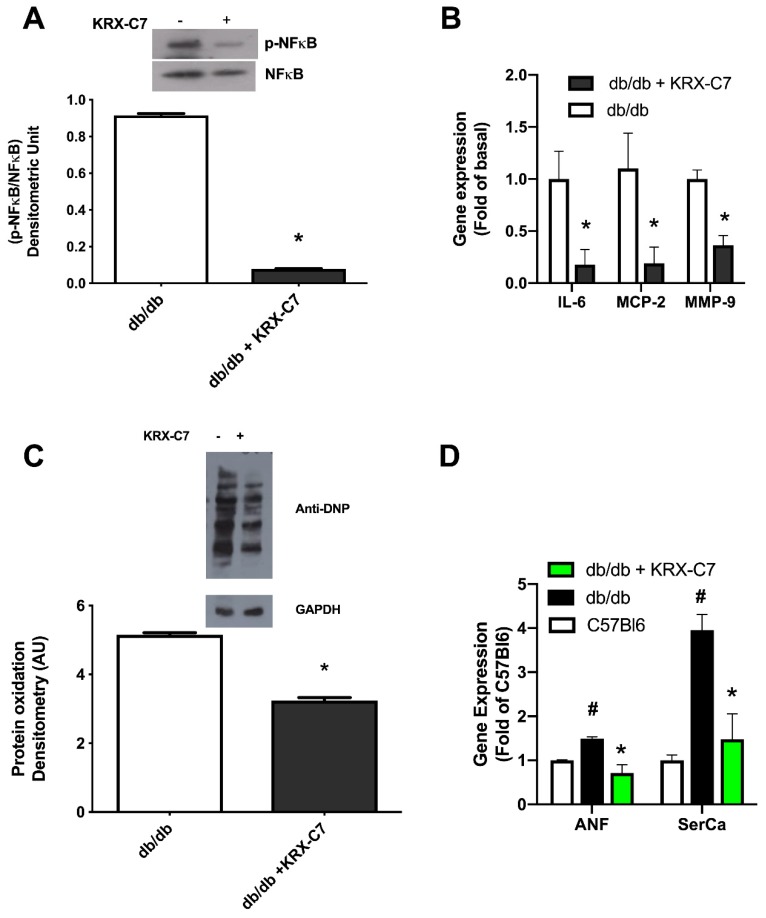
GRK2 inhibition reduces oxidative and hypertrophy responses in the diabetic heart. (**A**) Western blot analysis of phospho-NF-κB and total NF-κB levels on whole protein lysate from the cardiac tissue of KRX-C7 treated and control db/db mice (* *p* < 0.05). (**B**) Gene expression analysis of proinflammatory factors, IL-6, MCP-2, and MMP9, by real-time PCR on total RNA extract from the myocardium of KRX-C7 treated and control db/db mice expressed as fold change of control (* *p* < 0.05). (**C**) Levels of protein oxidation in cardiac tissue from KRX-C7 treated and control db/db mice shown as levels of 2,4-dinitrophenylhydrazine (DNP) incorporated into proteins. GAPDH was used as the loading control. The image is representative of three independent experiments. * *p* < 0.05. (**D**) Gene expression levels of ANF and SERCa2A, performed by real-time PCR on total RNA extract from the myocardium of C57Bl6 mice, db/db mice, and KRX-C7 treated db/db mice, expressed as fold of C57Bl6 (# *p* < 0.05 vs. C57Bl6, * *p* < 0.05 vs. db/db).

It is known that db/db mice recapitulate the diabetes induced cardiomyopathy (DCM) phenotype, including increased myocardial proinflammatory factors, such as NF-κB, and oxidative damage [27,28]. To evaluate the effects of KRX-C7 on DCM, we assessed the activation of NF-κB in the heart. The levels of phosphorylation of the p65 subunit were reduced in KRX-C7 treated db/db mice with respect to the vehicle-treated group (Figure 7A). Accordingly, the expression of related proinflammatory cytokines and chemokines, such as IL-6, MCP-2, and MMP9, was similarly reduced (Figure 7B). We also evaluated protein oxidation levels in the myocardium as a marker of oxidative damage and, interestingly, found it reduced as well (Figure 7C). Moreover, the treatment with KRX-C7 reduced Atrial Natriuretic Factor (ANF) and Sarco/Endoplasmic reticulum Calcium ATPase (SerCa) transcriptional levels (Figure 7D) in the myocardium, confirming the beneficial effects of this drug on DCM. 

## 3. Discussion

DCM remains one of the main problems in the management of diabetic patients and despite a growing interest in the pathophysiology of DCM, there are no specific guidelines for structuring a treatment strategy in clinical practice. Here, we propose a new antidiabetic target, GRK2, that can be inhibited experimentally with our HJ-loop custom peptide, KRX-C7. This peptide, beyond its effects on the glycaemic profile, exerts a potential therapeutic action on DCM. In particular, we showed that KRX-C7 is able to counteract the inflammatory phenotype of the diabetic heart by blocking the NF-κB dependent pathway. NF-κB is a key mediator of DCM development, as in the experimental model it was demonstrated that this factor is responsible for inflammatory and oxidative damage of the diabetic myocardium [29]. GRK2 represents an endogenous regulator of NF-κB, as the inhibition of the kinase reduces NF-κB activity in the hypertrophic heart [17]. Even though, in our setting, the effects of KRX-C7 on DCM could be a consequent of glycaemic metabolism and insulin sensitivity restoration, the known ability of GRK2 to regulate NF-κB suggests that the inhibition of the kinase through KRX-C7 could have a direct effect on DCM related inflammation. Indeed, it was demonstrated that pyrrolidine dithiocarbamate (PDTC), an inhibitor of NF-κB, produced beneficial effects on DCM in db/db mice without affecting glycaemic control [28]. 

G protein-coupled receptor kinase 2 (GRK2) is a critical regulator of the cellular transduction network. Upregulation of GRK2 has important effects in several cardiovascular conditions [7,8,30,31,32,33]. On the other hand, in recent years, several reports have demonstrated a key role for GRK2 in the modulation of insulin sensitivity in physiological and pathological conditions [15]. Indeed, GRK2 expression is increased in metabolic active tissues in different experimental models of IRES [13,14,34,35,36]. In particular, we have found that IRES is characterized by an enhancement of GRK2/IRS1 interaction, which reduces IRS1’s availability for insulin receptors, thus dampening insulin signaling [14]. Moreover, inhibition of GRK2 activity leads to increased insulin sensitivity in in vitro and in vivo models of insulin resistance, demonstrating that GRK2 mediates insulin resistance through a kinase-dependent mechanism [14,34,37]. These data propose GRK2 as a possible therapeutic target for metabolic disorders and that its inhibition may have therapeutic effects on all IRES related conditions, providing a new therapeutic approach for the treatment of T2DM [14,15]. 

Recently, we have identified KRX-C7, a novel GRK2 inhibitor, obtained through side chain cyclization of the linear peptide derived from fragment 383–390 of the HJ loop of GRK2 [23,24], which enhanced GRK2 inhibition properties, such as stability and selectivity, compared to parent linear molecules. The present study establishes that KRX-C7 improves insulin sensitivity and glucose homeostasis in vitro and in vivo, in an animal model of T2DM. 

The mechanism of KRX-C7 is the removal of the inhibitory effect of GRK2 on insulin signaling. The impact of KRX-C7 on insulin sensitivity is particularly effective as it involves the full cascade of phosphorylative events, from IRS1 to ERK and AKT, and leads to enhanced translocation of Glut4 on the plasma membrane and to glucose extraction. Intriguingly, we observed that KRX-C7 augments AMPK activation, which is an important cell energy sensor.

These findings are fully translated in vivo in db/db mice. KRX-C7 improved glucose and insulin tolerance. Similarly, the insulin signal transduction in db/db mice is also enhanced, and GRK2 inhibition profoundly affects the biochemistry of the diabetic skeletal muscle. Increased glucose utilization machinery in this tissue is probably responsible for the ameliorated removal of blood glucose in both IGTT and ITT. KRX-C7 administration increases the activation of signaling molecules, such as IRS, AKT, ERK, and GLUT4, in db/db treated mice. 

Our findings provide evidence that GRK2 can be an innovative therapeutic target for diabetes, with positive effects on diabetic cardiopathy. Indeed, heart failure remains a major cause of death and disability in diabetes. In our study, db/db mice present biochemical alterations typical of initial heart failure. In particular, SERCA2a protein levels are often observed to be reduced in the diabetic failing heart. This downregulation appears to be at the post-trasductional level, at least in the early stages, since increased SERCA2a mRNA is described in diabetic hearts [38]. Our data are in accordance with this literature, and in particular, the effect of KRX-C7 to reduce mRNA of SERCA2a confirms the maladaptive upregulation of SERCA2a in diabetes, and the effectiveness of GRK2 inhibition to correct such a feature.

Further exploration is required in multigenic models of diabetes, which are closer to human pathophysiology than db/db mice. Nevertheless, KRX-C7 holds promising translational features for the treatment of such a complex metabolic disease. 

## 4. Materials and Methods

### 4.1. Cell Culture

L6 myoblasts (CRL-1458, ATCC, Manassas, VA, USA) were maintained in DMEM medium supplemented with 10% fetal bovine serum. To allow differentiation into myotubes, FBS was restricted to 2% for 24 h. Cells were serum starved overnight in 0.5% DMEM/Bovine Serum Albumin (BSA) and treated for 15 min with insulin 10^−7^ mol/L FBS, with or without the GRK2 inhibitor, KRX-C7.

### 4.2. Cell Viability Assay

Cells were 96-well plates seeded to a density of 5000 cells/well. After 24 h, KRX-C7 was added at different concentrations for the different time points. At the end of incubation times, PrestoBlue™ Reagent (Invitrogen, Carlsbad, CA, USA) was added directly in the culture medium for 2 h, at 37 °C in the dark. Absorbance ratio 570/600 nm was used to quantify viability. Results were expressed as the percentage relative to vehicle-treated control (0.5% DCM was added to untreated cells).

### 4.3. KRX-C7 Internalization.

L6 myoblasts were 16-well plated (10,000 cells/well) and serum starved overnight. Cells were then incubated with fluorescently labeled peptides (F-KRX-C7, 10^-6^ mol/L) for 60 min at 37 °C. After washing twice with PBS, cell images were taken by using an Eclipse E1000 Fluorescence Microscope Nikon Instruments Europe BV and acquired by using Sigma Scan Pro software (Jandel, San Jos, CA, USA). Images were optimized for contrast in Adobe Photoshop (Adobe, San Jose, CA, USA), but no further manipulation was made.

For fluorescence quantification, cells were 24-well-plated (20,000 cells/well), serum starved overnight, and incubated with fluorescently labeled peptides (F-KRX-C7) at concentrations of 10 and 10^−6^ mol for 60 min at 37 °C. Incorporated fluorescence was quantified with a 485 nm excitation filter and a 510 nm emission filter using a gain setting of 1.0 nm on a plate reader (Genios, Tecan, Mamedorf, Switzerland). 

### 4.4. Deoxy-Glucose Uptake Measurement

L6 cells were twelve-well plated (5 × 10^5^/well) in 10% FBS supplemented DMEM. Cells were then starved with DMEM containing 25 mM glucose and no serum at 37 °C. After 12 h of serum deprivation, the medium was removed and replaced with lower glucose DMEM (5 × 10^−3^ mol/L) for a further 12 h. Quiescent L6 myoblasts were pretreated with KRX-C7 (100 nM) for 30 min and then stimulated with Insuline (10^-7^ mol/L) for 30 min. Glucose transport was measured as (3H)-2D-deoxyglucose (2-DG, 0.5 μCi/mL) uptake for 10 min in triplicates. The reaction was stopped by medium removal, cells washed 3 times with ice cold PBS, and once with ice-cold 20% trichloroacetic acid followed by solubilization with 1 N NaOH. Incorporated radioactivity was determined by liquid scintillation. Aliquots from each well were used to determine protein concentration using the bicinchoninic acid-Assay (BCA) (Pierce-Thermo Scientific, Rockford, IL, USA).

### 4.5. GRK2 Activity Assay

To evaluate the effect of KRX-C7 on GRK2 activity, we assessed GRK2 activity by light-dependent phosphorylation of rhodopsin-enriched rod outer segment membranes (ROS) using [γ-32P]-ATP as previously described [23,39]. Briefly, L6 myoblast or db/db skeletal muscle were homogenized in ice-cold detergent-free lysis buffer (25 × 10^−3^ mol/L Tris-HCl (pH 7.5), 5 × 10^−3^ mol/L EDTA, 5 × 10^−3^ mol/L EGTA, 1 × 10^−3^ mol/L phenylmethylsulfonyl fluoride). Cytosolic fractions and membrane fractions were separated by centrifugation. Soluble GRK activity was assessed in cytosolic fractions (100–150 µg of protein), which were incubated with ROS membranes in the presence or absence of KRX-C7 in reaction buffer (25 μL; 10 × 10^−3^ mol/L MgCl_2_, 20 × 10^−3^ mol/L Tris–HCl, 2 × 10^−3^ mol/L EDTA, 5 × 10^−3^ mol/L EGTA, and 0.1 × 10^−3^ mol/L ATP and 10 μCi of [^γ32^P]-ATP). After incubation on white light for 15 min at room temperature, the reaction was quenched with ice-cold lysis buffer and centrifuged for 15 min at 13,000× *g*. The pellet was washed twice in ice-cold lysis buffer to remove the unbound [^γ32^P]-ATP and then resuspended in 100 μL of buffer and the [^γ32^P]-ATP incorporation was determined by a liquid scintillation counter.

### 4.6. Animals 

All animal procedures were performed in accordance with the policies and guidelines of the “Position of the American Heart Association on Research Animal Use” [40] and were approved by the Ethics Committee of the “Federico II” University of Naples (2009/86653, 25/6/2009). The European Commission Directive 2010/63/EU was followed. Six month old male db/db mice were implanted with an intraperitoneal osmotic pump to deliver KRX-C7 (5 mg/kg/day) or NaCl for 15 days. Blood pressure was measured in a non-invasive manner as previously described [14]. Three age and sex matched C57bl6 mice were used as a wild type referral for the cardiac gene expression study.

### 4.7. Glucose Tolerance Test

Fasted overnight mice were subjected to a glucose tolerance test (GTT) through intraperitoneal glucose injection (2 g/kg i.p) [41]. Blood glucose levels were measured by tail bleeding (Glucose Analyzer II; Beckman Coulter, Brea, CA, USA) at indicated time points [41]. 

### 4.8. Insulin Tolerance Test

Fasted mice were injected IP with insulin (0.75 IU/kg body weight). Blood glucose was measured by tail bleeding at 0, 15, 30, 60, and 120 min after insulin injection. 

### 4.9. Ex Vivo Glucose Uptake 

2-Deoxyglucose (2-DOG) transport was measured in skeletal muscle as previously described. Isolated whole femoral quadriceps muscles were incubated in flasks containing oxygenated Krebs-Ringer-Bicarbonate and then subjected to insulin treatment. Subsequently, samples were transferred to a second flask and incubated at 37 °C for 10 min in Krebs-Henseleit Buffer containing 1 × 10^−3^ mol/L [3H]-2-D-deoxyglucose (3 mCi/10^−3^ mol/L; Perkin Elmer, Boston, MA, USA). Next, muscle samples, washed in ice-cold PBS and dissolved in NaOH, were subjected to liquid scintillation counting for the dual labels, and the extracellular and intracellular spaces calculated to determine glucose uptake. Radioactivity in the supernatant was measured using a scintillation counter (Beckman Coulter, Brea, CA, USA).

### 4.10. Insulin Signaling

For the insulin signaling studies in vivo, fasted or fed mice were injected intraperitoneally with insulin at 10 U.I./kg [42]. After 15 min, mice were sacrificed and tissues were collected and stored at −80 °C until processing.

### 4.11. Immunoprecipitation and Western Blot 

Frozen tissues or L6 myoblasts were homogenized with a Polytron (Brinkman Instruments, Riverview, FL, USA) in ice-cold RIPA/SDS buffer (50 × 10^−3^ mol/L Tris-HCl (pH 7.5), 150 × 10^−3^ mol/L NaCl, 0.01 g/L NP-40, 0.0025 g/L deoxycholate, 2 × 10^−3^ mol/L Na3VO4, 0.2 g/L sodium, 2 × 10^−3^ mol/L EDTA, 2 × 10^−3^ mol/L PMSF). For plasma membrane isolation, samples were homogenized in Tris (pH 7.5 25 × 10^−3^mol/L)/edta (5 × 10^−3^ mol/L)/egta (5 × 10^−3^ mol/L) buffer. Lysates were separated into aliquots, snap-frozen in liquid nitrogen, and stored at −80 °C. In all experiments, lysis buffers also contained phosphatase inhibitors (Sigma-Aldrich, Saint Lois, MI, USA). Protein concentrations were determined using the BCA assay kit (Pirce, Rockford, IL, USA) or with the Quick Start Bradford Protein Assay (Bio-Rad, Hercules, CA, USA). Endogenous total lysates were immunoprecipitated with selective antibody and protein G plus/protein A-conjugated agarose beads (Santa Cruz Biotechnology, Dallas, TX, USA) overnight at 4 °C. Samples were washed twice with lysis buffer, twice with phosphate-buffered saline, and resuspended in SDS gel loading buffer and subjected to Western blotting. The following specific antibodies were used: Total Insulin Receptor β subunit (1:1000; Santa Cruz Biotechnology, Dallas, TX, USA), GRK2 (1:1000; Santa Cruz Biotechnology, Santa Cruz, CA, USA), total IRS1 (1:1000; Cell Signaling Technology, Danvers, MA, USA), Phospho-IRS1 (Tyr 989; 1:1000; Santa Cruz Biotechnology, Santa Cruz, CA, USA), total AKT (1:1000; Santa Cruz Biotechnology, Santa Cruz, CA, USA), phospho-AKT (Ser 473; 1:1000; Cell Signaling Technology, Danvers, MA, USA), total GSK3α (1:1000, Cell Signaling Technology, Danvers, MA, USA), Phospho-GSK3α (Ser21; 1:1000; Cell Signaling Technology, Danvers, MA, USA), Actin (1:1000; Santa Cruz Biotechnology, Dallas, TX, USA), Glut 4 (1:2500 Abcam, Cambridge, MA, USA) ERK1/2 (1:1000; Cell Signaling Technology, Danvers, MA, USA), Phospho-tyrosine 42/44 ERK (1:1000; Cell Signaling Technology, Danvers, MA, USA), AMPK (1:1000; Cell Signaling Technology, Danvers, MA, USA), Phospho-Ser272-AMPK (1:1000; Cell Signaling Technology, Danvers, MA, USA), Actin (1:1000; Santa Cruz Biotechnology, Santa Cruz, CA, USA), Gαs (1:1000; Santa Cruz Biotechnology, Santa Cruz, CA, USA). Blots were visualized by enhanced chemiluminescence (ECL-plus detection kit, GE Healthcare Life Science, Bukinghamshire, UK) and quantified by using Image Quant TL software (GE Healthcare Life Science, Bukinghamshire, UK).

### 4.12. RNA Extraction and Real Time PCR

The levels of genes expression were determined by real-time reverse transcription polymerase chain reaction (rRT-PCR) on three samples for each experimental group. RNA extraction was performed by TRIzol Reagent ready to use (Invitrogen, Carsbad, CA, USA) starting from 50 mg of cardiac tissue. The amount of isolated RNA was dissolved in 50 µL of RNAse-free water and the concentration was determined by a micro-Volume spectrophotometer (MaestroGen, Carson City, NV, USA). 2 µg of RNA for each sample was used as a template for reverse transcription (RT) performed by a One-Step RT-PCR kit (Applied Biological Material-abm, Richmond, BC, Canada) containing random primers mixed in a total volume reaction of 20 µL. The rRT-PCR was performed using a StepOne Real-Time PCR System-Applied Byosistem (Thermo Scientific, Rockford, IL, USA) by SyberGreen as the identification method, and in each amplification tube, a total volume reaction of 20 µL was composed by: 20 ng of synthetized cDNA (in a volume of 2 µL), 10 µL of BrightGreen 2X qPCR MasterMix-ROX (Applied Biological Material-abm, Richmond, BC, Canada), and 2 µL of forward and reverse primers in a final concentration of 500 nM and up to volume with nuclease free water (Applied Biosystems, Foster City, CA, USA). Each tube was prepared in triplicates, and the RT-qPCR assay for 18 s. IL-6, MMP-9, MCP-2, ANF, and SerCa2A were performed with the primers listed in Table 1. Thermal cycling started with an initial denaturation step at 95 °C for 5 min. After this initial step, 40 cycles of PCR were performed. Each RT-PCR cycle consisted of heating at 95 °C for 15 s, 60 °C for 30 s for annealing, and 72 °C for 1 min for the extension. At the end of the reaction, melting curve analysis was performed to evaluate the specificity of the amplification reaction for each primer pair. The levels of 18 s mRNA was used for normalization [43]. The gene expression levels for each target gene was determined using the comparative Ct method normalized for 18 s and showed as fold changes in mRNA levels of the treated group relative to the control group. 

### 4.13. Protein Oxidation 

The detection of carbonyl groups introduced into proteins by oxidative reactions occurring inside cells was performed using OxyBlot-Protein Oxidation Detection Kit (Millipore, Burlington, MA, USA), by following the manufacturer’s protocol. Briefly, 5 μL containing 15 μg of proteins from cardiac lysate was derivatized through a reaction with 2,4-dinitrophenylhydrazine (DNPH), which converts the carbonyl groups of proteins to 2,4-dinitrophenylhydrazone (DNP-hydrazone). The DNP-derivatized protein mixtures were analyzed by Western blot analysis [44]. 

### 4.14. Statistical Analysis

Each experiment was performed in triplicate to ensure reproducibility. All data are presented as mean ± SE. Statistical differences were determined by one-way or two-way ANOVA as appropriate, and Bonferroni post hoc testing was performed when applicable. A *p* value < 0.05 was considered significant. Statistical analysis was performed using GraphPad Prism (version 6.01; GraphPad Software Inc., San Diego, CA, USA).

## 5. Conclusions

Our paper provides the evidence that GRK2 inhibition can be safely achieved through means of engineered peptides targeting the HJ loop of the kinase, with beneficial effects in diabetes. Glucose metabolism is indeed ameliorated, and so it is the CDM observed in db/db mice. This is the first observation of the beneficial use of GRK2 inhibitors in cardiac alterations in diabetes. 

## Figures and Tables

**Table 1:** List of primers used in Real Time PCR.

Genbank Accession #	Gene	Sense Primer Sequence	Antisense Primer Sequence
19791	18s	GTAACCCGTTGAACCCATT	CCATCCAATCGGTAGTAGCG
16193	IL-6	GAGGATACCACTCCCAACAGACC	AAGTGCATCATCGTTGTTCATACA
17395	MMP9	CTTCTGGCGTGTGAGTTTCCA	ACTGCACGGTTGAAGCAAAGA
230899	ANF	ACCTGCTAGACCACCTGGAG	CCTTGGCTGTTATCTTCGGTACCGG
20296	MCP-2	GGGTGCTGAAAAGCTACGAG	TCCAGCTTTGGCTGTCTCTT
